# Dose escalated intensity modulated radiotherapy in the treatment of cervical cancer

**DOI:** 10.1186/s13014-015-0551-0

**Published:** 2015-11-24

**Authors:** Nikola Cihoric, Alexandros Tsikkinis, Coya Tapia, Daniel M. Aebersold, Inti Zlobec, Kristina Lössl

**Affiliations:** Department of Radiation Oncology, Bern University Hospital & University of Bern, Bern, Switzerland; University of Bern, Institute for Pathology, Murtenstrasse 31, Bern, 3010 Switzerland; Department of Translational Molecular Pathology, University of Texas MD Anderson Cancer Center Life Science Plaza, 2130 W. Holcombe, Blvd. Unit 2951, Houston, 77030 TX USA

**Keywords:** Cervical cancer, Chemo radiation, Dose escalated radiotherapy, IMRT

## Abstract

**Purpose:**

Standard dose of external beam radiotherapy seems to be insufficient for satisfactory control of loco-regionally advanced cervical cancer. Aim of our study is to evaluate the outcome as well as early and chronic toxicities in patients with loco-regionally advanced cervical cancer, treated with dose escalated intensity modulated radiotherapy (IMRT) combined with cisplatin chemotherapy.

**Material and methods:**

Thirty-nine patients with cervical carcinoma FIGO stage IB2 – IVA were treated with curative intent between 2006 and 2010. The dose of 50.4 Gy was prescribed to the elective pelvic nodal volume. Primary tumors < 4 cm in diameter (*n* = 6; 15.4 %) received an external beam radiotherapy (EBRT) boost of 5.4 Gy, primary tumors > 4 cm in diameter (*n* = 33; 84.6 %) received an EBRT boost of 9 Gy. Patients with positive lymph nodes detected with ^18^FDG-PET/CT (*n* = 22; 56.4 %) received a boost to a total dose of 59.4 - 64.8 Gy. The para-aortic region was included in the radiation volume in 8 (20.5 %) patients and in 5 (12.8 %) patients the para-aortic macroscopic lymph nodes received an EBRT boost. IMRT was followed with a 3D planned high dose rate intrauterine brachytherapy given to 36 (92.3 %) patients with a total dose ranging between 15–18 Gy in three fractions (single fraction: 4–6.5 Gy). Patients without contraindications (*n* = 31/79.5 %) received concomitantly a cisplatin-based chemotherapy (40 mg/kg) weekly. Toxicities were graded according to the common terminology criteria for adverse events (CTCAE v 4.0).

**Results:**

Mean overall survival for the entire cohort was 61.1 months (±3.5 months). Mean disease free survival was 47.2 months (±4.9 months) and loco-regional disease free survival was 55.2 months (±4.4 months). 65 % of patients developed radiotherapy associated acute toxicities grade 1, ca. 30 % developed toxicities grade 2 and just two (5.2 %) patients developed grade 3 toxicities, one acute diarrhea and one acute cystitis. 16 % of patients had chronic toxicities grade 1, 9 % grade 2 and one patient (2.6 %) toxicities grade 3 in the form of vaginal dryness.

**Conclusion:**

Dose escalated IMRT appears to have a satisfactory outcome with regards to mean overall survival, disease free and loco-regional disease free survival, whereas the treatment-related toxicities remain reasonably low.

## Introduction

The last major advancement in the treatment of cervical cancer was made more than 15 years ago. Several large prospective randomized clinical trials have shown that the combination of radiotherapy with chemotherapy prolongs the disease-free survival while also reducing mortality. Even though the outcome of radio-chemotherapy is better than that of radiation alone, the combined regimen was associated with a higher incidence of side effects while loco-regional control and overall survival still remained unsatisfactory [1–4]. Between twenty to forty percent of patients treated with conventional radiotherapy will relapse loco-regionally, not only outside of the treatment field but also within the treated volume [5]. Unfortunately, the prognosis for these patients is very poor with only a few therapeutic options available such as pelvic exenteration or palliation. The reasons for the loco-regional failure may be attributed to geographical target miss, insufficient dose of radiation delivered to the primary tumor and/or to the nodal area [5, 6].

At the same time, a rapid development of the radiation delivery machines and cancer imaging has been achieved. These changes are well-mirrored in a significantly improved therapeutic ratio of IMRT for various tumor entities, including cervical cancer [7]. External beam dose escalation results in more favorable treatment outcome while the incidence of radiation induced toxicities, early and chronic, is sinking. Incorporation of multimodal imaging, especially in patients with cervical cancer, enhances the detection of nodal or systemic disease, and leads to a better definition of local disease [6].

However the level of evidence in the utilization of IMRT and of new imaging modalities, in the treatment of gynecological malignancies is still low. There is currently only one phase II prospective randomized trial, comparing conventional radiotherapy to IMRT, with published results [8]. In the available literature, there are many suggestions and different approaches in the treatment of cervical cancer using highly conformal radiotherapy and yet there is still no well-defined concept concerning the required total dose, the volume and the required constraints. Furthermore, even though there is a known potential benefit on quality of life, as well as a favorable toxicity profile of IMRT compared to 3D conformal radiotherapy, there is still an ongoing debate about its general use in gynecological cancer. In addition, there is still not enough data available, concerning the possibility of dose escalation using external beam radiotherapy for the treatment of both the primary tumor as well as nodal disease [9].

In 2006, our group began using IMRT as a standard radiation delivery method for cervical cancer patients. Our treatment concept was based on usage of escalated external beam radiotherapy dosage for treating the infiltrated regional lymph nodes and of the primary tumor compared to the standard dosage (45-50Gy). We conducted this retrospective study in order to evaluate the treatment-related toxicities, early and chronic, as well as the clinical outcome of cervical cancer patients treated with escalated dose of IMRT.

## Material and methodes

### Patients

Patients with histologically confirmed cervical cancer, FIGO stage IBII to IVA, treated with IMRT at the Bern University Hospital Department of Radiation Oncology were included in this retrospective study according to the institutional ethical standards. We evaluated all medical and radiotherapy records, pretreatment and follow-up images of 39 patients treated between October 2006 and January 2011. Patient characteristics are summarized in Table 1. Median follow-up time was 35 months.

**Table 1:** Patient characteristic

Age at time of diagnosis: median (range)	59.5 year (range: 26 to 89)	
	No.	%
FIGO stage		
IB2	4	10.3
IIA	2	5.1
IIB	20	51.3
IIIB	9	23.1
IVA	4	10.3
Size of primary tumors		
Tumors < 4 cm	6	15.4
Tumors > 4 cm	33	84.6
Lymph nodes status		
N0	15	38.5
N1	24	61.5
Paraaortic Positive LN	5	12.8
Median number of positive LN per patient	2.5 (range: 1 to 5)	
Tumor histology		
	No	%
Adenocarcinoma	9	23.1
Squamous Cell Carcinoma	30	76.9
Tumor grade		
Grade 1	1	2.6
Grade 2	25	64.1
Grade 3	13	33.3

Before treatment all patients underwent pretreatment staging workup, including medical history, general physical and gynecological examination, digital rectal examination, tumor biopsy, comprehensive laboratory analysis and in cases of suspected rectal or bladder infiltration, additional rectoscopy and cystoscopy. The primary tumor was clinically assessed by an experienced gynecologist and radiation oncologist. Tumor staging was defined according to the International Federation of Obstetrics and Gynecology (FIGO) and the TNM-UICC system. In addition, local tumor extension was assessed by MRI, but the initial tumor stage was not influenced by the findings. All patients with cervical cancer clinically staged as IB2-IIB underwent pretreatment staging with ^18^FDG-PET/CT scan. If the PET-CT revealed, clear signs of metastatic lymph nodes, the patients were selected for definitive radio-/chemotherapy. In case of negative PET-CT findings, patients were selected for surgery, were a sentinel lymph node dissection was performed with frozen section pathological evaluation. If the sentinel lymphadenectomy revealed positive lymph nodes, or an advanced tumor stage was intraoperatively detected, the surgery was stopped and the patients were referred for definitive radio-/chemotherapy. If the sentinel lymphadenectomy revealed no positive lymph nodes, a complete surgery was performed, including a paraaortal lymphadenectomy. Any further therapy decisions were made in a multidiscipline tumor board setting after complete pathological evaluation. Patients with clinical stage FIGO IIB or greater were selected for radio-chemotherapy. In cases with doubtful or suspicious findings regarding lymph node status those were surgically evaluated.

At the beginning of 2010 our policy was changed regarding surgical staging, and all patients, regardless of tumor stage, were surgically evaluated (*n* = 11). Two (5.1 %) patients have positive lymph nodes that were not previously detected by imaging and 3 (7.7 %) patients have advanced local tumor extension. These patients subsequently received definitive radio-chemotherapy. Information on surgery and concomitant therapy data are shown in Table 2.

**Table 2:** Therapy data

Surgery and concomitant therapy		
	n	%
Patients with incomplete (primary) surgery	4	10.3
Patients with surgical nodal staging	11	28.2
Clinical nodal staging	28	71.8
Complete chemotherapy (Cisplatin 40 mg/m2 weekly)	31	79.5
Patients without all cycles of chemotherapy due to hematological toxicities	3	7.7
Patients without chemotherapy due to contraindication or refusal	5	12.8
Radiotherapy dosage		
	Median (Gy)	Range (Gy)
Radiotherapy duration (days)	60	46 - 96
EBRT elective pelvic nodal dose (Gy)	50.4	45-55.8
EBRT elective paraaortral dose (Gy)	47.7	45 - 50.4
EBRT Tumor Boost dose (Gy)	9	5.4 - 21.6
EBRT Total Tumor Dose (Gy)	59.4	50.4 - 72
LN Boost dose (Gy)	62	59.4 - 64.8
Brachytherapy total dose (Gy)	18	10 - 24
Brachytherapy single dose (Gy)	6	5-6

### Radiotherapy planning: treatment volumes and dose prescription

All patients underwent a planning computer tomography (CT) without contrast in supine position. Patients were instructed to come for the planning CT as well as the daily radiotherapy fractions with a full bladder. No particular instructions were given regarding rectal filling. The CT slice thickness was 3 mm. A personalized adjusted immobilization device was created for each patient.

Image sets acquired by CT, diagnostic ^18^FDG-PET/CT and MRI were imported into the Eclipse Planning System (Varian Medical System, Palo Alto, CA). We used the “automatic matching algorithm”, with manual correction as needed. Registration quality was regarded as acceptable, when bony structure misalignment did not exceed </=1 mm. External beam radiotherapy was delivered using a dynamic multi-leaf linear accelerator with a photon energy of 6 MV.

The gross tumor volume of the cervix (GTVc) was defined as the visible macroscopic tumor based on all the available clinical and imaging data. Clinical target volume for primary tumor area (CTVc) encompassed GTVc, uterus, parametria and the upper third of the vagina. In cases of vaginal involvement, CTVc expanded 2 cm into the vagina caudally of the tumor. The planning target volume of primary tumor (PTVc) was created using anisotropic expansion, considering cervical and surrounding structure movements. The PTVc was expanded to 15 mm in the antero-dorsal direction and 10 mm in the lateral direction. PTVc was manually corrected if needed. The asymmetrical margin for PTV was chosen based on the fact, that in cervical cancer, movement is not uniform in all directions [10]. In the dorsal direction PTVc margin extended maximally to the posterior rectal wall and in frontal direction maximally 1.5 cm into the bladder.

In the first phase PTVc was irradiated with a total dose of 50.4 Gy. After 45 Gy a control MRI was performed to evaluate tumor response and measure tumor size. In cases where the remaining tumor was larger than 4 cm in diameter, an additional EBRT boost of 9 Gy was administered to the PTVc. Otherwise, for tumors smaller than 4 cm in diameter, the PTVc was irradiated with an EBRT boost of 5.4 Gy. Single dose was 1.8 Gy.

### Nodal PTV and SIB volume

The elective clinical target lymph nodes (LN) volume encompassed the common, external and internal iliac lymphatic chain to the aortic bifurcation and presacral LN area. In case of LN involvement at the level of the common artery or aortic LN, we extended the elective nodal volume to the level of the renal arteries. A safety margin of 7 mm was added to define the nodal planning target volume (PTVn). PTVc and PTVn were merged to one single planning target volume (PTVsum).

Nodal gross tumor volume (GTVn) was based on data acquired by ^18^FDG-PET/CT including assessment of other imaging modalities (CT, MRI). Positive LNs were delineated separately as nodal gross tumor volume (GTVn). PTVn boost was formed by adding a safety margin of 5 mm to the GTVn. A total of 22 (56.4 %) patients were given a nodal boost, 12 (30.8 %) with consecutive and 10 (25.6 %) with simultaneous integrated boost (SIB). Rational and methods for SIB delivery are described in our previous work [11].

**Fig. 1:**
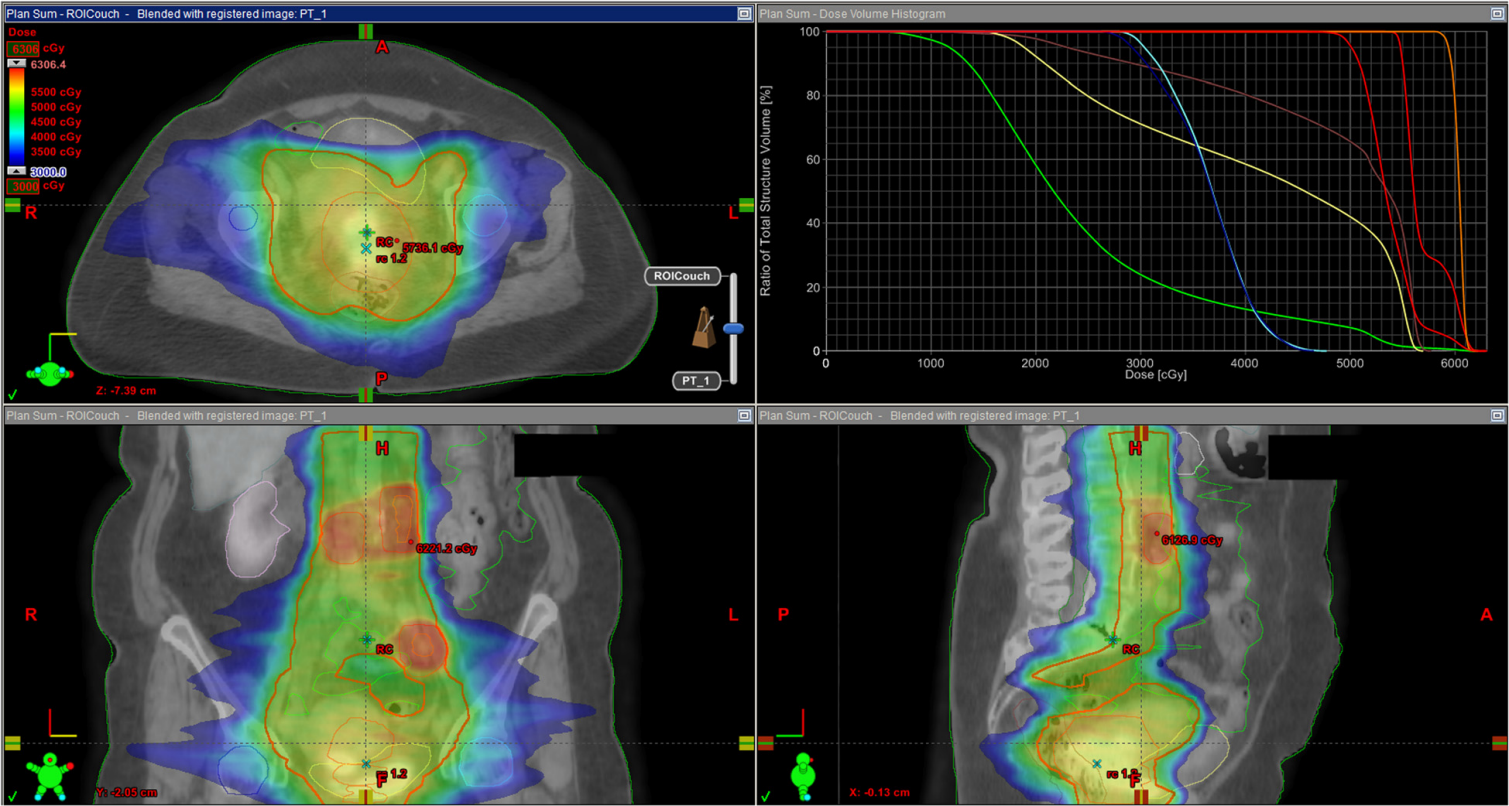
Example of treatment plan

An example of a treatment plan is shown in Fig. 1.

### Constraints for organs at risk

Organs at risk were delineated on the axial CT slices. We delineated the rectum up to the sigmoid. The bowel was contoured to the level extending one vertebral body beyond the upper border of the PTV, including large and small intestines. The bladder and femoral heads were also contoured. Dose constraints for organs at risk were standardized as follows: 60 % of rectal volume should receive not more than 50 Gy, 35 % of bowel volume should receive no more than 35Gy, 50 % of bladder volume should receive no more than 50 Gy and 10 % of the femoral heads volume should receive no more than 50 Gy.

### Brachytherapy

EBRT was followed by a HDR boost to the primary tumor in 36 (92.3 %) patients, administered one week after completion of EBRT. Brachytherapy consisted of a total dose between 15 to 18 Gy delivered in 2 to 4 weekly fractions, with a single dose of 4 to 6.5 Gy. We used a microSelectron® HDRB Unit and a Vienna Ring CT-MRI Applicator Set. MRI images were evaluated together with our radiology department to define the high and intermediate risk areas. Afterwards, we reconstructed the high risk and intermediate risk areas detected in the MRI images on our planning CT images. Planning volumes were planned according to the GEC-ESTRO guidelines [12]. Because of the dose escalation of EBRT we adapted our brachytherapy dose constraints as suggested in the ABS guideline [13]. Three patients (7.7 %) refused the administration of a brachytherapy boost.

### Chemotherapy

Patients were scheduled to receive concomitant cisplatin based chemotherapy (40 mg/kg) weekly during the full course of radiotherapy. 31 (79.5 %) patients received the chemotherapy as planned. The details for the remaining patients (n: 8, 20.5 %) are listed in Table 2.

### Toxicities assessment and follow-up

We assessed patients for acute toxicities weekly during the course of radiotherapy and once 6 weeks after therapy completion. Toxicities were graded according to the CTCAE v 4.0. The initial tumor response was evaluated clinically by a gynecological oncologist 3 months after radiotherapy with a follow up 3 months thereafter for the next two years, and every 6 months afterwards. Clinical evaluation includes a PAP smear. We conducted an ^18^FDG-PET/CT 6 months after therapy to evaluate the local control as well for the detection of systemic metastases, as recommended in the NCCN guidelines.

Failure was defined as persistent disease or tumor recurrence following radiotherapy. The date of failure was defined as the date of any signs of disease, either clinical or by imaging. The site of failure was recorded as local, nodal (regional) and distant. Furthermore, a distinction was made between in-volume nodal failures or “out of volume” nodal failures. Moreover, we distinguished between nodal failures in the boost region from failure in the elective volume.

### Statistical consideration

Primary endpoints of interest were overall survival (OS, time between the first day of radiotherapy to the date of death, independent of cause, or the date of the last follow-up), disease-free survival (DFS, time between the first day of radiotherapy to the date of any sign of tumor relapse) and loco-regional disease-free survival (LRDFS, time between the first day of radiotherapy to the date of any sign of tumor relapse in the former tumor bed or the nodal CTV region). Differences in survival time were analyzed using the log-rank or Wilcoxon’s test and plotted using Kaplan-Meier curves. P-values <0.05 were considered statistically significant. All analyses were carried out using SAS (V9.2; The SAS Institute, Cary, NC).

## Results

### Recurrence rate and site of recurrence

During follow-up 25 (64.1 %) patients had complete response three months after therapy and remained disease free within follow-up. Median follow-up time was 35.5 months. Fourteen (35.9 %) patients developed a recurrence. One (2.6 %) patient developed isolated local relapse and one (2.6 %) patient isolated nodal relapse. Nine (23.1 %) patient developed loco-regional recidive [8 (20.5 %) local and 5(12.8 %) nodal]. All but, two loco-regional recidive was within treated volume. Twelve (30.8 % of all patients, or 85.7 % of patients with relapse) patients developed systemic disease. Isolated systemic disease occurred in 5 (12.8 %) patients.

The pattern of recurrence follows in the majority of patients the natural course of cervical cancer, where the loco-regional relapse occurs first, followed by systemic metastasis in 5 patients. Two patients had simultaneously loco-regional and systemic relapse and only 1 patient had systemic metastasis preceding loco-regional failure. Median time to relapse at any site was 9 months (range 3 to 38 months), with median time to systemic metastasis being 12 months (range 3 to 38 months) and loco-regional metastasis being 9 months (range 5–25 months).

We did not record any failures in the regions of positive lymph nodes treated with boost (consecutive or SIB).

### Survival time

OS, DFS and LRFS were analysed and stratified by FIGO stage, tumor grading (G1/G2 versus G3), number of positive lymph nodes and tumor size (cut-off 4 cm).

**Fig. 2:**
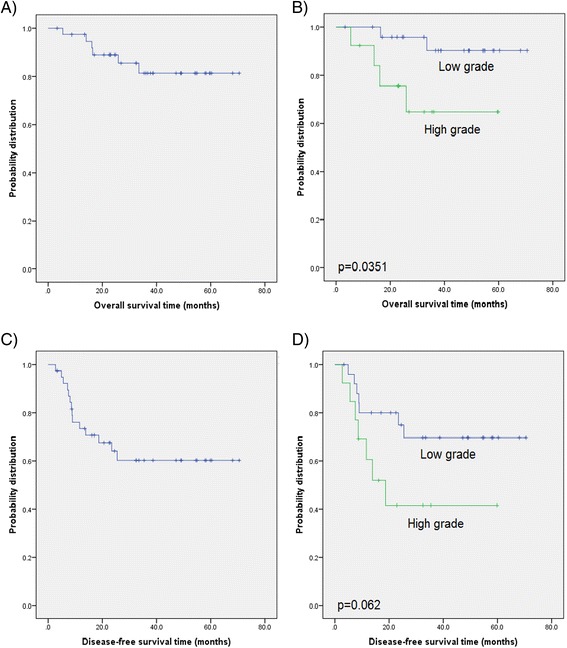
Kaplan-Meier curves of **a**) overall survival time for the entire cohort followed by **b**) stratification by tumor grade, **c**) Disease-free survival time for the entire cohort and **d**) stratified by tumor grade (log-rank test)

In terms of OS (5-year OS was 76.3 %), there was no association with FIGO stage (p = 0.771), number of positive lymph nodes (p = 0.9173) or tumor size (p = 0.301) although a statistically significant difference was observed in patients with a higher tumor grading (p = 0.035; Fig. 2a and b).

5-year DFS was 60.3 %. There was no statistically significant relationship between FIGO stage and DFS (p = 0.3937) or tumor size (p = 0.283). Patients with higher tumor grade tended toward a worse DFS than their low tumor grade counterparts (p = 0.0619, Fig. 2c and d), while no difference in survival time was found for patients with a high or low number of affected lymph nodes (p = 0.3597).

No association of FIGO (p = 0.739), tumor size (p = 0.65), tumor grade (p = 0.778), or lymph node positivity (p = 0.691) was observed in terms of LRFS (5-year LRFS was 75.1 %).

### Toxicities

There were no acute or chronic toxicities greater than grade 3 and we did not record any toxicity related treatment breaks.

The most common acute toxicity was diarrhea, recorded in 15 (38.5 %) patients, followed by vaginal mucositis in 12 (30.8 %), cystitis in 9 (23.1 %) and proctitis in 7 (17.9 %) patients. The majority of acute side effects were grade 1 and 2, self-limited or treated with dietary interventions or symptomatic therapy. One (2.6 %) patient experienced acute diarrhea grade 3 and one (2.6 %) acute cystitis grade 3. All acute toxicities were resolved within 6 weeks after radiotherapy.

The most common chronic toxicity was vaginal stricture recorded in 8 (20.5 %) patients followed by cystitis in 3 (7.7 %) patients; one (2.6 %) patient developed a chronic proctitis grade 2. An overview of the acute and late toxicities is summarized in Table 3.

**Table 3:** Overview of early and late toxicities

	Acute: *n* (%)				Chronic: *n* (%)			
Toxicities grade	Small bowel	Rectum	Bladder	Vagina	Small bowel	Rectum	Bladder	Vagina
0	24 (61.5)	32 (82.1)	30 (76.9)	27 (69.2)	39(100.0)	38 (97.4)	36 (92.3)	31 (79.5)
1	9 (23.1)	4 (10.3)	6 (15.4)	7 (17.9)	0 (0.0)	0 (0.0)	1 (2.6)	3 (7.7)
2	5(12.8)	3 (7.7)	2 (5.1)	5 (12.8)	0 (0.0)	1 (2.6)	1 (2.6)	4 (10.3)
3	1 (2.6)	0 (0.0)	1 (2.6)	0 (0.0)	0 (0.0)	0 (0.0)	1 (2.6)	1 (2.6)
4	0 (0.0)	0 (0.0)	0 (0.0)	0 (0.0)	0 (0.0)	0 (0.0)	0 (0.0)	0 (0.0)
5	0 (0.0)	0 (0.0)	0 (0.0)	0 (0.0)	0 (0.0)	0 (0.0)	0 (0.0)	0 (0.0)

## Discussion

The experience with IMRT in the treatment of cervical cancer is limited and there is currently only one phase II prospective randomized trial, comparing conventional radiotherapy to IMRT, with published results [8], Even less is known about external beam dose escalation using IMRT for this tumor entity.

The outcome and toxicities of conventional external beam radiotherapy combined with concomitant chemotherapy, is well summarized in the meta-analysis of the “*Meta-Analysis Group, Medical Research Council Clinical Trials Unit*” [14]. The group analyzed individual data from 13 trials, comparing concomitant radio chemotherapy versus chemotherapy alone in patients with cervical cancer FIGO stage IB to IVA. Delivered dose ranged from 40 Gy to 45 Gy to the whole pelvis with EBRT boost in the primary tumor region up to 61.2 Gy and consecutive low dose rate or HDR brachytherapy boost from 18 to 50 Gy. The combined regime showed an improvement in the overall DFS and the loco regional DFS of 8 % and 9 % at 5 years respectively. Disease free survival at 5 years was 58 %. The benefit in survival was accompanied by higher rates of serious adverse events including gastrointestinal toxicities.

Compared to conventional or 3D radiotherapy, IMRT achieves a better conformity and sparing of normal tissue. Roeske et al. showed a reduced dose to the small bowel [15]. Several other authors showed a good target coverage with a significantly lower dose to the bladder, rectum, and small bowel [16–18]. Ahmed et al. and Esthappan et al. introduced a more aggressive approach for patients with metastatic paraaortic lymph nodes while sparing risk structures and achieving an acceptable level of early and chronic unwanted events. Dosimetric studies were followed by several retrospective analyses, where authors showed a favorable toxicities profile of IMRT (Table 4) [19–23].

**Table 4:** Literature overview

Author and year of publication	Therapy seetings and study type	IMRT EBRT total dose/EBRT daily dose	Brachytherapy total dose	Number of patients treated with IMRT	FIGO Stage	Number of patients with positive pelvic lymph nodes	Number of patients with negative lymph nodes	Follow-up (median) in months	Total number of patients with disease relapse	Loco-regional failure	DFS/patient alive without sign of disease	OS/alive at last follow up	Acute gastrointestinal or genitourinary toxicites Gr 3 or greater	Chronic gastrointestinal or genitourinary toxicites Gr 3 or greater	Normal tissue planning constraints
*Gerszten et al.,* [23]	Definitive, extended field IMRT, retrospective	45 Gy and 55 Gy to involved nodes	25 Gy/5 Gy	22	IB-IIIB	9 (receiving nodal boost) 2 positive on surgical staging	11	No data	No data	No data	No data	No data	0	Not evaluated	rectal maximum dose: 54 Gy with no more than 40 % of the rectal volume receiving 40 Gy; bladder maximum dose 54 Gy with no more than 50 % at 40 Gy; small bowel maximum 50 Gy with no more than 35 % of small bowel receiving 35 Gy.
*Kidd et al.*, [6]	Definitive 18 FDG PET based IMRT, retrospective comparison with non-IMRT radiochemotherapy	50 Gy to whole pelvis and additional 20 Gy to central region (cervix)	6 weekly fraction of 6.5 Gy HDR	135	IBI-IVA	41 (30.4 %)	68 (50.4 %)	22 (range, 5–47 months)	39	11 (8.1 %)	91 (67.4 %) pts	No data	No data	8 (6 %)	<40 % of bowel to receive 30 Gy, <40 % of rectum to receive 40 Gy, <40 % of pelvic bones to receive 40 Gy, and <40 % of femoral heads to receive 30 Gy.
*Hasselle et al.,* [25]	Definitive IMRT in 81 patients. Retrospective multicentric study.	median 45 Gy (range: 39.6 - 50.4 Gy)/1.8 Gy	LDR 35 to 40 Gy; 5 HDR fractions to 27.5 to 30 Gy	111 (22 postop, 8 with consecutive surgery)	I–IVA	No data	No data	26.6 (range, 5.4–99.0 months)	No data	The 3-year pelvic failure rate - 29.2 %	69 % (95 % CI, 59–81 %)	78 % (95 % confidence interval [CI], 68–88 %)	2 % (95 % CI, 0–7 %)	7 % (95 % CI, 2–13 %)	rectum: maximum dose < 50 Gy; bowel V45 < 250 mL, pelvic BM V20 < 75 %, V10 < 90 %; bladder: as low as reasonably achievable
*Chen et al.,* [26]	Definitive, IMRT, retrospective	45–54 Gy, (54–60 nodes simultan boost)	HDR 20 – 33.5 Gy/4–6 Gy/Fraction 2 x Week	109	IB2 - IVA	14	82	32.5 for survival pts (5–75)	5 (4.6 %) locoregional only; 14(12.8 %) distant only; 29 (26.6 %) in total;	21.9 % at 3 year	67.6 % at 3 year	78.2 % at 3 year	3 pts (GI Only)	5 (4.6 %) GI 7 (6.4 %) GU	rectum: V30 < 50 %; small bowel: V30 < 15 %;
*Du XL et al.,* [29]	Definitive RT-CH. Comparison of reduced field IMRT with conventional EBRT.	30 Gy to whole pelvis with additional boost of 30 Gy to lymphatic drainage region as well as paracervix and parametrium.	HDR 10–30 Gy/5–6 Gy SD	60	IIB–IIIB	No data	No data	7 months (range, 6 - 68 months)	64.90 %	No data	64.9 % PFS at 5 y	82.5 % at 3 y; 71.2 % at 5 y	7	0	No data
*Gandhi et al.* [8]	Definitive radio chemotherapy. Nonblind, prospective, randomized, phase II trial. Comparison with whole pelvis conventional radiotherapy	50.4 Gy/1.8 Gy	21 Gy/7 Gy SD	22	IIB-IIIB	No data	No data	21.6 months (range, 7.7-34.4 months).	5 (22.7 %)	2 (9.1 %)	60 % at 27 months	85.7 % at 27 months	2	0	small bowel: volume receiving 40 Gy (V40) <32 %, maximum dose <50 Gy; rectum: V40 < 40 %, maximum dose <50 Gy; bladder: V40 < 40 %, maximal dose < 50 Gy
*Jensen LG et al.*, [31]	Definitive, extended-field intensity-modulated radiotherapy	45 to 50.4, median boost dose to parametrian: 9 Gy or pelvic LN 10 Gy in 16 pts, PA boost of median 10.4 Gy in 6 pts	LDR 35 to 40 Gy 1 or 2 x; HDR 19.8 to 30 3 to 5 x	21	IB1 - IIIB	14 patients had paraaortic LN and 20 pelvic LN	0	22 (range, 12 to 56 months) for survived patients	11	No data	42.9 % (95 % CI, 26.2 % Y70.2 %). At 11 months	59.7 % (95 % confidence interval [CI], 41.2 % Y86.4 %) at 11 months	4(19 %)	2 (9.5 %)	rectum: maximum dose < 50 Gy; bowel: V45 < 250 cm3; bladder: as low as reasonably achievable.
*Cihoric et al*. [11]	Definitive dose escalated IMRT, retrospective	50.4 to whole pelvis, 5.4 to 9 Gy boost to central disease, 62 Gy to lymph nodes	HDR TD 15–18 Gy with 4–6.5 Gy SD	39	IB2 to IVA	24 (61.5 %)	15	36 (3–71 months)	14 (35.9 %)	9 (23.1 %) patient with pelvic failure; LRFS was 55.2 ± 4.4 months	25 (64.1 %). The mean DFS: 47.2 ± 4.9 months	Mean OS time for the entire cohort: 61.1 ± 3.5 months	2 (5.2 %)	2 (5.2 %)	60 % of rectum < 50 Gy, 35 % of bowel < 35Gy, 50 % of bladder < 50 Gy and 10 % of the femoral heads < 50 Gy.

Traditionally, EBRT whole-pelvic doses are limited to 45 to 50 Gy, primarily due to the small bowel tolerance as a limiting factor. As already discussed, IMRT has an improved therapeutic ratio and therefore introduces the possibility of sparing healthy organs without a compromise to the target volume dosage. Incidence of early grade 3 or higher gastrointestinal (GIT) and urogenital (UG) toxicities for conventional radio-chemotherapy is more than 25 % [3, 24]. Compared with conventional radiotherapy overall incidence of toxicity in our cohort was low. We did not record any serious adverse event grade 4 or 5, and despite of the EBRT dose intensification, acute toxicities in our patient cohort remain within reasonable range. A similar incidence of serious adverse events ranging between 2 % to 4.6 % for acute and 6.4 to 7 % for late toxicities is reported by other authors. A review of the literature with their corresponding results is presented in Table 4 [8, 25–31].

In our institution we defined our treatment concept based on two goals. Firstly, by using an escalated dose we tried to sterilize the macroscopic metastatic disease in the lymphatic pathways while reducing the primary tumor size, which in turn had a benefit for the application of the brachytherapy boost. We managed to achieve treatment results, comparable with results reported in the literature.

Kidd et al. compared outcome and toxicities of 317 cervical cancer patients treated with step-wedge intensity modulation technique and 135 patients treated with PET/CT-guided IMRT. The majority of patients were diagnosed with FIGO IIB and IIIB. 30.4 % and 17.0 % of patients treated with IMRT were diagnosed with metastatic pelvic and para-aortic lymph nodes respectively. The IMRT group showed better disease specific survival and overall survival [6].

Hasselle et al. treated 81 patients (FIGO IIB-IVA) with definitive IMRT followed by HDR or LDR brachytherapy. The prescription dose for IMRT ranged between 39.6 and 50.4 Gy with median dose of 45 Gy. Three-year OS and the DFS were 61.4 % and 51.4 %, respectively. The 3-year pelvic failure rate (PF) was 29.2 %. The 3-year cumulative incidences of PF alone, DF alone, synchronous PF/DF, and CM as first events were 8.6 %, 10.1 %, 4.8 %, and 7.0 %, respectively. Six patients had isolated PF, and five had synchronous PF and DF. For all patients, the 3-year cumulative incidence of PF was 13.6 %. Pelvic failure locations included the cervix in 5 patients, vagina in 4 patients, vulva in 1 patient, and cervix plus a pelvic lymph node in 1 patient [25].

Chen et al. conducted a retrospective study with 109 patients with cervical cancer (FIGO IB2-IVA). Positive pelvic LN and PALN were diagnosed in 12.8 % and 11.9 % respectively. IMRT dose given to the GTV ranged from 45 to 54 Gy with concomitant boost of 54 to 60 Gy to the involved lymph nodes. The median follow up time for all surviving patients was 32.5 months, with a range from 5 to 75 months. The 3-year OS, LFFS (local failure free survival) and DFS were 78.2 %, 78.1 % and 67.6 %, respectively [26]. The treatment concept used, was similar to ours.

The major difference in patient population between the cited studies and ours is seen in the incidence of detected nodal metastasis and patients with bulky tumors > 4 cm. A significantly lower percentage of patients were diagnosed with metastatic lymph nodes in contrast to our cohort (61.5 % with lymph nodes metastases). Even though a review of the available literature would suggest a worse outcome in lymph node positive patients, this was not the case in our patient collective [32]. It could be hypothesized that an efficient control in the pelvic region contributed to the overall disease control. An important result of our study is the fact that we have achieved an excellent control of the metastatic lymph nodes with a median dose of 62 Gy [range 59.4 to 64 Gy]. During follow-up, there was no nodal failure detected within the high dose nodal volume.

However, 8 patients developed loco-regional recidive within irradiated volume and one patient outside irradiated volume in form of distant vaginal failure. Reasons for failure cannot be precisely determined in this moment. Several potential factors, such as tumor resistance, geographical miss or insufficient dose, are stated in literature. In our cohort potential reason for local failure can be insufficient brachytherapy dose. In early stages of IMRT technique adoption we were cautious due to potential excess of toxicity due to escalated external beam dose. Our median summed EQD2 dose was 77.1 Gy (calculated using the formula: EQD2 = BED/(1 + 2/α/β), where BED = ED* (1 + ED/α/β) and α/β = 10). At the time of our protocol development there were not much data about combined escalated dose EBRT with HDR BT. However, this dose may be not sufficient for treatment of bulky tumors. Due to findings of our interim institutional analysis we changed policy and from 2011 onwards our dose prescription for HDR brachytherapy was 4 × 6 Gy prescribed to high risk tumor volume as defined by GEC-ESTRO guideline [12].

Further possible reasons for therapy failure are geographical miss or failure to detect gross disease, either local or nodal, and irradiate with sufficient dose. Although we used three imaging modalities including clinical evaluation, it is possible that we did not succeed to recognize all area needed to be irradiated with high dose. Every imaging modality has some limitation to detect local or nodal disease. However, the most appropriate staging is a topic of an ongoing debate. Although, surgical staging is more reliable than CT based staging and several retrospective studies have shown an advantage of surgical node extirpation, there is still no high level evidence to incorporate surgical staging or therapy of bulky lymph nodes into clinical practice. This must be observed in the context of more advanced imaging and radiotherapy techniques. Although not perfect, PET-CT appears to be a valid addition to clinical staging of patients with cervical cancer [33, 34]. Extensive lymphadenectomy combined with radio chemotherapy may be connected with excess toxicity and bulky lymph nodes may be treated with advanced IGRT technique [11]. Prospective clinical trials are required to answer this questions.

Even though, lymph node involvement is one of the most important prognostic factors in cancer, evaluation and staging is not well defined in cervical cancer patients [35]. In addition several questions remain open regarding planning volumes and up to date there is still no clear concept regarding nodal PTV. A recent retrospective analysis of 665 primarily operated patients, revealed 168 patients with nodal metastases, with the most common site of occurrence, being the obturator and iliacal nodal stations, were affection of other nodal region was rare. These findings may help for further future optimization of the PTV [36].

Almost all patients with a loco-regional failure have a systemic failure as well. It is still unclear, whether metastatic cancer cells are already present at the time of therapy begin or if they are a result of seedling from tumor recurrence. A potential therapeutic answer for the treatment of microscopic systemic disease and at the same time, for a better loco-regional control, could be the introduction of an additional chemotherapeutic agent to cisplatin. Duenas-Gonzales et al. showed encouraging results, by combining gemcitabine with cisplatin, but a broader adoption of that concept was abandoned, as a result of the higher incidence of toxicities [37]. A dual chemotherapy concept should be evaluated in the future, together with IMRT as radiation delivery method.

It should also be noted that our study has several limitations. The small patient cohort prevents us from reaching a valid statistical conclusion in regards to possible risk factors and the therapy outcome. At the same time, there is no comparison to an additional arm comparing a different treatment dose. Furthermore, our cohort is heterogeneous, with some of our patients being partially operated prior to administering chemoradiation. Late toxicities of the proposed treatment concept are also an important issue. Late gastrointestinal toxicities occur in ca. 10 % of all patients and most occur within the first two years, but can still emerge up to 20 years after treatment. The urological toxicities can rise up to 10 % and their incidence can increase over time. Our relatively short follow-up is limited in the detection of potential late toxicities [38].

We managed to show that the intensified radiation therapy is well tolerated by patients with advanced cervical cancer; however we could not show survival benefit, potentially as a result of our small patient cohort. Therefore, the results of this study have to be validated on a larger patient cohort in order to show its impact on recurrence rate and survival. Nevertheless, our study can be used for new treatment strategies for patients with loco-regionally advanced cervical cancer.

## Conclusion

Patients with loco-regionally advanced cervical cancer treated with intensified IMRT seem to have a satisfactory outcome with reasonably low levels of treatment related toxicities. Although being limited due to its small size and retrospective nature, the present study contributes to the notion that the application of a high dose of radiation in the pelvic region by means of IMRT is feasible, with an acceptable profile of unwanted events and good loco-regional control, comparable with other published studies.

## Abbreviations

3D: three-dimensional
18FDG-PET/CT: F-18-fluordeoxyglucose positron emission tomography
CT: computed tomography
CTVc: clinical target volume for primary tumor area
DFS: disease-free survival
EBRT: external beam radiation therapy
FIGO: international federation of gynecology and obstetrics
GTVc: gross tumor volume of the cervix
GTVn: nodal gross tumor volume
IMRT: intensity modulated radiotherapy
LRDFS: loco-regional disease-free survival
LN: lymph nodes
MRI: magnetic resonance imaging
OS: overall survival
PTVc: planning target volume of primary tumor
PTVn: nodal planning target volume
PTVsum: single planning target volume
SIB: simultaneous integrated boost
